# The League of Mercy

**Published:** 1901-02-23

**Authors:** 


### THE LEAGUE OF MERCY.

A public meeting of the Fulham branch of this league was held in the town-hall on February 12th, Colonel A. A. Owen presiding. There was an excellent gathering, and amongst those supporting the chairman on the platform were Sir Henry Burdett, Mrs. Harrison (lady president of the Fulham branch), Mr. Frank Manby, Mr. R. Butler, and others

The Chairman opened the proceedings by proposing that the meeting should formally express its profound sorrow at the great loss sustained by the whole nation, and more particularly by the League of Mercy and kindred societies, by the death of our late beloved Queen. He hoped they would never forget the example of Her Majesty, and that they would always remember that the League of Mercy as a branch of the Prince of Wales's Hospital Fund was very near her heart. He himself had had the honour and pleasure of being associated in the work, as the president of the Fulham branch, with one of the best lady presidents it would be possible to find, and he was happy to say that their work had been most successful. In the first year of their existence, for example, the collection had realised £247, and in the second year it had sprung to £409. In introducing Sir Henry Burdett, Colonel Owen spoke of the great honour done to the branch by so notable a member of the League having consented to give an address.

Sir Henry Burdett said it gave him very great pleasure to be able to address the members of the branch and their friends, because it was evident that Fulham had its heart in the work. It occupied a very honourable and proud position in the League of Mercy, standing second on the list of the purely metropolitan divisions and occupying a very prominent position amongst the whole of the 110 branches. Sir Henry then went on to speak of the quiet, unostentatious work of our late Queen—a work entirely free of that self-advertisement which was one of the curses of the age. In quietness and confidence lay the strength of the individual, and in quietness and confidence also lay the strength, the endurance, and the reality of the Empire. From this he drew the moral of the immense importance of personal service in all philanthropic work. Proceeding to deal more particularly with the hospital question, he stated that the number of patients treated in the voluntary hospitals of London had increased by 400,000 during the past ten years. Something like £180,000, or within £20,000 of the whole sum represented by this increase, had been raised by means of the Hospital Sunday and Saturday Funds and latterly the Prince of Wales's Hospital Fund. This being so, they might ask, What need is there of the League of Mercy? He answered that it was perfectly true that sufficient money had been raised to provide the needed accommodation, but to this day there was a debt of £100,000 on the hospitals of London, which it was the object of the League to help to wipe out. It existed for the purpose of extending the area of subscribers and of inducing small contributors to become annual subscribers, anyone being eligible for membership who would undertake to get twenty other people to each give one shilling during the year. In dealing with the institution and organisation of the League Sir Henry referred to the practice of giving hospital tickets. He understood that this old bugbear still existed in Fulham. He could not understand how anyone of ordinary intelligence could attach any value to these tickets, which could only serve as introductions to the hospitals, and could not secure admission, seeing that a patient was only admitted on the merits of his case. In conclusion, the speaker referred to the absolute necessity of hospitals as a means of medical training, and to the great superiority of voluntary over State institutions of the kind.

Mr. Frank Manby, in proposing a hearty vote of thanks to Sir Henry Burdett for his address, said that the League of Mercy had undoubtedly succeeded in obtaining subscriptions from those persons who would otherwise not feel able to give anything, but it was a much more difficult matter to reach to any large extent those who actually used the hospitals. This latter was one of the objects of the League, and its accomplishment called for much care and trouble. But they must remember that it was more blessed to give than to receive, and that no gift was blessed to the giver unless it had entailed some self-denial.

Mrs. Harrison, who seconded the motion, said she was sure a fresh and stronger interest would be felt in the League as a result of Sir Henry Burdett's address, and that that renewed interest would be visibly shown by the acquisition of many recruits, who would be most warmly welcomed.

The vote was heartily accorded.

At this point Mr. Peter Lawson, having obtained permission to put a question, asked Sir Henry Burdett whether the money collected by the League in Fulham would in any way benefit the local hospital at Fulham.

Sir Henry Burdett replied that the funds were apportioned only amongst those hospitals with reference to which entirely satisfactory reports were received from the visitors appointed by the League, and no other claims were considered.

Mr. Butler, who proposed a vote of thanks to the Chairman and to Mrs. Harrison, said they ought to be very grateful to Colonel Owen for arranging the meeting. He had done more for the borough of Fulham than anyone else he (the speaker) knew. He was a man under whom everyone could work with pleasure, and who knew how to smooth over all friction.

Sir Henry Burdett seconded, and the motion was carried unanimously.

A number of new members of the League were afterwards enrolled.

